# The Long-Term Effects of Short-Period Adalimumab Biosimilar Usage in Ankylosing Spondylitis

**DOI:** 10.7759/cureus.36444

**Published:** 2023-03-20

**Authors:** Arvind Chopra, Nagnath Khadke, Manjit Saluja, Toktam Kianifard, Anuradha Venugopalan, Mihir Gharia

**Affiliations:** 1 Department of Rheumatology, Center for Rheumatic Diseases, Pune, IND; 2 Medical Content and Services, Digicare Health Solutions Private Limited, Ahmedabad, IND

**Keywords:** drug toxicity, treatment, clinical practice, biosimilar, ankylosing spondylitis, adalimumab

## Abstract

Background

Cost and drug toxicity frequently deter the long-term use of anti-tumor necrosis factor (TNF) agents in ankylosing spondylitis (AS). Therefore, this study was conducted to observe long-term relief after the short-term administration of an anti-TNF agent.

Methodology

A one-year, prospective, interventional, uncontrolled, single-center trial was conducted. There were 50 patients with symptomatic active chronic AS who received rheumatology therapy and were anti-TNF naive. Every two weeks, 40 mg of standard biosimilar adalimumab (Bs-ADA, Exemptia™) was administered subcutaneously for six injections (10 weeks) or to continue with standard follow-up if they did not achieve an Assessment in Ankylosing Spondylitis Response Criteria (ASAS 20) index response by week 12. Standard indicators (Assessment Spondyloarthritis International Society/ASAS and Bath) were used to evaluate progress. In addition, TNF-alpha, interleukin (IL)-6, and IL-17 were tested using a commercially available enzyme-linked immunosorbent assay kit from Bio Legend (Bengaluru, India).

Results

Patients experienced early and significant improvement in pain, non-steroidal anti-inflammatory drugs (NSAIDs) requirement, function, and several indices (ASAS 20 and 40, ASAS partial remission, Bath Ankylosing Spondylitis Disease Activity Index, Bath Ankylosing Spondylitis Functional Index, Ankylosing Spondylitis Disease Activity Score) after discontinuing injections. At weeks 12 and 48, 84% and 52% of patients showed ASAS 20 improvement, with 34% and 24% showing ASAS partial remission. Over half of the patients continued to improve and provided proof of concept.

Conclusions

In difficult-to-treat AS, a 10-week course of biosimilar adalimumab demonstrated significant early improvement that often lasted for 24 weeks. This unconventional method proved to be economically appealing. It merits further confirmation and acceptance, especially in resource-constrained contexts.

## Introduction

Ankylosing spondylitis (AS) is an autoimmune inflammatory painful disorder of the axial skeleton and sacroiliac joints that causes significant limitations in spine motion, spine deformity, systemic complications, and early mortality [1]. The earlier detection and evaluation of skeletal damage are now possible because of more recent imaging modalities such as magnetic resonance imaging (MRI) and computed tomography (CT) [2]. AS is a long-term disorder that is difficult to treat [3]. Non-steroidal anti-inflammatory drugs (NSAIDs) are the cornerstone of symptomatic treatment. Conventional disease-modifying anti-rheumatic drugs (DMARDs) may or may not be effective. However, biological DMARDs and anti-tumor necrosis factor (TNF) drugs are more effective and are recommended for long-term use [3].

The prevalence of AS in India has been reported to be 0.03% (95% confidence interval (CI) = 0.02, 0.05), implying a significant disease burden [4]. However, the high cost of biological DMARDs and the risk of infections such as tuberculosis (TB) continue to be a barrier to their widespread use in India [5].

Several biosimilar anti-TNF drugs have been launched worldwide to improve access and affordability [5,6]. Adalimumab (Humira™) is a popular anti-TNF medication that is not available in India. Adalimumab (Exemptia™, Bs-ADA) was marketed as a standard biosimilar in India in 2014 [7,8]. A donation of Exemptia™ made it possible for the investigator to initiate the current study, which is non-commercial and not-for-profit.

The author was inspired to experiment with a new concept after repeatedly observing prolonged symptomatic relief following a shorter (non-standard) treatment period with infliximab or etanercept in several patients with AS in clinical practice. A preliminary report was presented at the 2005 Asia Pacific League of Associations for Rheumatology (APLAR) Congress [9]. Short-term therapy appeared to be more effective, safer, and more acceptable to patients [9]. This study was designed with this viewpoint in mind.

The goal of this study was to assess the long-term efficacy and safety of a short-term regimen of biosimilar adalimumab in the treatment of active chronic symptomatic AS.

## Materials and methods

This was a prospective, interventional, uncontrolled, single-center study that lasted for a year. From June 2016 to July 2018, the study was conducted in a popular community rheumatology clinic (Center for Rheumatic Diseases (CRD), Pune, India). The protocol was developed in accordance with the principles outlined in the most recent version of the Declaration of Helsinki published in 2013. Furthermore, it was sanctioned by the local ethics committee (CRD, Pune) and was registered (CTRI/2018/02/012018) [10].

Inclusion criteria included (i) clinical diagnosis of AS made by a rheumatologist according to the Assessment in SpondyloArthritis International Society (ASAS) criteria; (ii) patients who were at least 18 years old and no older than 75 years old; (iii) patients who had symptomatic disease, and patients who met any two of the following three criteria during the previous week: (a) axial muscular-skeletal pain with a Visual Analog Scale (VAS) score of 3 cm or greater (range = 0-10 cm) for more than three months, (b) early morning stiffness lasting 30 minutes or more, (c) an erythrocyte sedimentation rate (ESR, Westergren method) of 28 mm end of the first hour or C-reactive protein (CRP) assay of less than 6 mg/L; (iv) bony erosions and/or ankylosis as seen on a plain digital standard skiagram of the pelvis for sacro-iliac joints; (v) patients who need frequent NSAIDs being on a stable dose for at least four weeks; and (vi) duration of disease of fewer than two years.

The following were considered to be exclusion criteria: (i) arthritis and/or pain in the axial spine from any cause other than reactive arthritis; (ii) use of biological agents (especially anti-TNF) in the previous two years; (iii) use of DMARDs (except sulfasalazine and/or methotrexate) in the previous 12 weeks; (iv) use of steroids in the previous four weeks; (v) recent onset of a potentially serious medical disorder (such as cardiovascular disease or diabetes); (vi) history of any recent infection that required antibiotics in the last 12 weeks; (vii) history of past tuberculosis, no matter how it was treated; (viii) history of the human immunodeficiency virus (HIV) or hepatitis; (ix) history of cancer or lymphoproliferative disease; and (x) exposure to any type of hazard or risk because of participation in the study, as judged by the investigator.

Potential patients were found at the outpatient clinic (CRD, Pune), where a comprehensive clinical database has been in place since 1998. Patients were evaluated for eligibility and enrolled on a first-come, first-served basis after signing the informed consent form. Following enrolment, patients returned to the study site (CRD) every two weeks for the first 10 weeks to receive the Bs-ADA injection (total of six injections), and then at 12, 24, 36, and 48 weeks (one-year study completion).

According to the protocol, 40 mg Bs-ADA was injected subcutaneously every two weeks. Patients who did not achieve an ASAS 20 index response by week 12 were randomly assigned to either two additional fortnightly injections (Bs-ADA) or to continue with routine follow-up. BS-ADA was never repeated after that. Patients were given advice on how to manage their pain and how to take NSAIDs. They were given a daily therapeutic dose of NSAID for the first four weeks, then switched to a need-for-response (PRN) basis, as determined by the rheumatologist.

The majority of patients were already taking etoricoxib before they were enrolled in the study (60 mg and 90 mg tablets, India Pharmacopeia), and they were allowed to continue doing so (initially between 60 and 120 mg daily) after they were enrolled.

Other NSAIDs could be used, but the dose had to be converted to etoricoxib equivalent for the purpose of compiling the mean daily NSAID dose data. For this, 1,000 mg naproxen, 150 mg diclofenac, 100 mg indomethacin, or 900 mg etodolac were determined to be equivalent to 120 mg etoricoxib. Any and all forms of steroid use were outlawed. Patients’ medications for any other coexisting diseases were continued in consultation with their primary care physician.

A standard rheumatology case record form was used, as well as other standard AS questionnaires, including those recommended by ASAS [2,11-14]. In addition, the patients filled out the Indian adaptation of the modified Stanford Health Assessment Questionnaire (HAQ) [15]. Among the important measures were the following: (i) VAS was used to record musculoskeletal pain (the most severe pain felt in the previous 24 hours), patient and physician global assessment (current), and general health (current horizontal line with anchors at 0 for no value and 10 for the highest possible value was what it appeared to be); (ii) a numeric scale rating (NSR) on a horizontal scale with 10 distinct categories (1-10); (iii) Bath Disease Activity Index (BASDAI) with six questions on the NSR to assess symptoms from the previous week, including fatigue, neck-back-hip pain, pain/swelling in peripheral joints, discomfort when eliciting local tenderness (entheses), duration and severity of early morning stiffness, and other symptoms; (iv) Bath Functional Index (BASFI) with 10 NSR items for daily physical activities; (v) the primary efficacy variable was the ASAS 20 index improvement response, which evaluated four domains: patient global assessment, overall pain, function (BASFI), and inflammation (mean of BASDAI questions 5 and 6 pertaining to morning stiffness); achieved with a minimum 20% improvement with at least 1-unit improvement in NSR in three of the four domains without worsening in the remaining domain (20% or more and more than 1 unit); (vi) ASAS 40 index improvement response: domains similar to ASAS 20, but requiring 40% improvement, a minimum 2-unit NSR improvement, and no worsening; (vii) ASAS partial remission required none of the four ASAS 20 domains to exceed 2 on the NSR; (viii) ASAS 5/6 required a 20% improvement in five of the six domains (ASAS 20 domains plus domains of lateral spine flexion and CRP assay); (ix) a standard online programme was used to calculate the Ankylosing Spondylitis Disease Activity Score (ASDAS), which included total back pain, patient global disease activity, peripheral painful/swollen joint, morning stiffness, and ESR.

Patients were required to arrive at the study site in a fasting state (no meal or study drug intake) during the scheduled visit (8-11 am). In the case report form (CRF), adverse events (AE) were recorded. In accordance with the protocol, patients were given laboratory examinations during the screening process, as well as at the baseline and weeks four, eight, 12, 24, 36, and 48 (one-year) endpoints. These included a routine hemogram, blood sugar, metabolic hepatic and renal profile, urine analysis, ESR (Westergren method, normal range: less than 20 mm for men and less than 30 mm for women at the end of the first hour), and CRP titer (nephelometry, normal range 0- 5 mg/dL). To determine whether or not a patient had latent tuberculosis (TB), standard skin tuberculin testing (5 tuberculin unit (TU) intradermal), blood TB (gamma interferon release assay), and skiagrams of the chest were utilized [16]. TNF-alpha, interleukin (IL)-6, and IL-17 were tested using a commercially available enzyme-linked immunosorbent assay (ELISA) kit from Bio Legend (Bengaluru, India).

The biosimilar Adalimumab (Exemptia™) was provided free of charge to 13 patients and at a nearly 50% concessional market rate to the remaining study participants. Patients were responsible for their own medication costs, purchasing NSAIDs, and analgesics. All investigations were free of charge. Patients were each given a meager sum of money to help cover the costs of travel and food.

A convenient (non-probabilistic) sample size was chosen for statistical design and data analysis based on local resources, with an emphasis on at least 35 participants. Based on previous experience, a 20% dropout rate was predicted. SPSS version 2007 (IBM Corp., Armonk, NY, USA) was used to perform appropriate statistical tests, with significance set at two-sided p-values of <0.05. The mean, median, SD, proportions, and 95% confidence interval (CI) were calculated for the quantitative variables. At the end of the week, an intention-to-treat analysis was carried out (the last observation carried forward). At the week 12, 24, 36, and 48 endpoints, ASAS 20 was the primary efficacy response.

The proof of concept for the current study was accepted if at least 50% of the patients who had an ASAS 20 response at week 12 (two weeks after the Bs-ADA treatment was stopped) showed that their response persisted for another 24 weeks (at week 24 and week 36 endpoints).

A conventional survival analysis, also known as the life table method, was performed. The term *survival* was used to refer to the absence of an event (such as an ASAS 20, ASAS 40, ASAS 5/6 response, or an ASAS partial response) for a period of at least eight weeks in this study.

## Results

**Figure 1:**
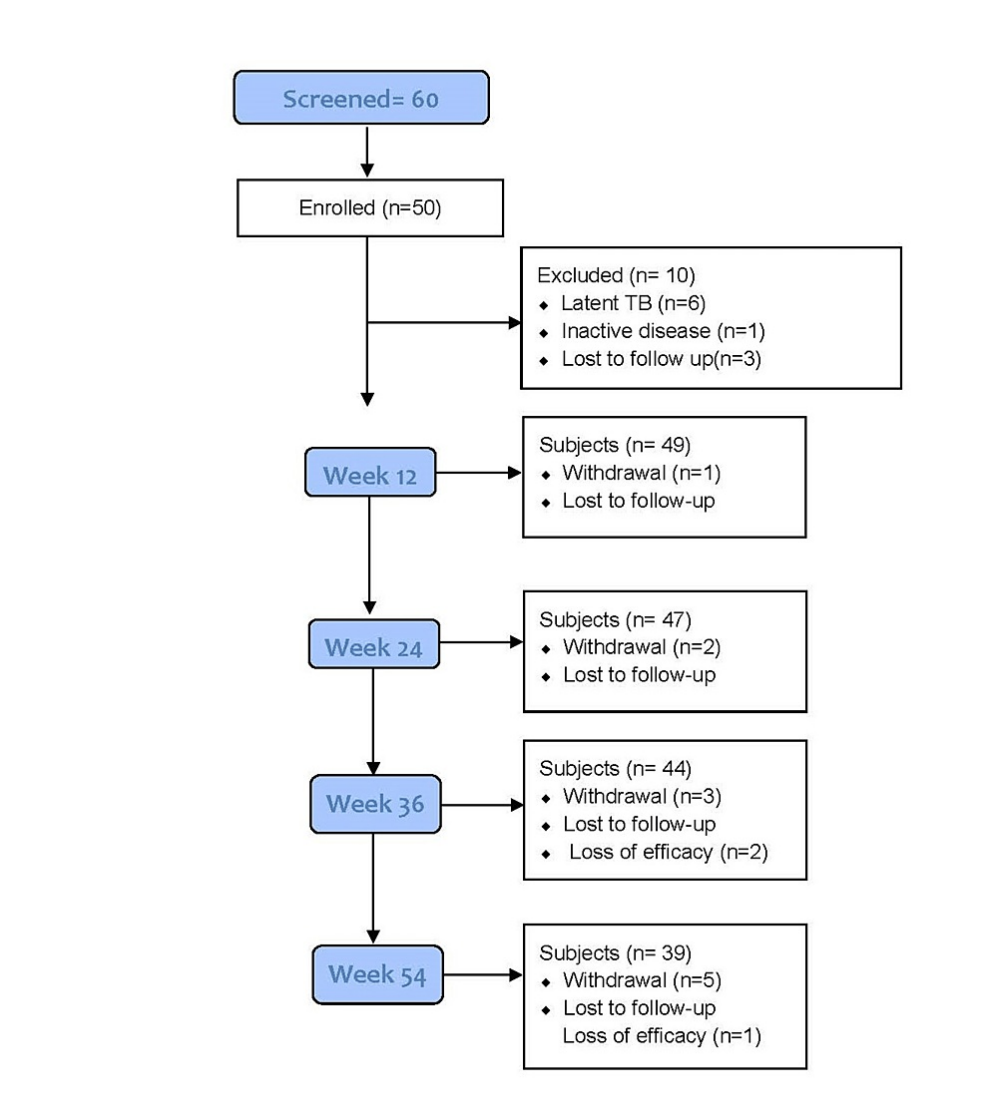
Patient disposition consort diagram. TB: tuberculosis

After screening 60 patients, 50 (42 men and 8 women) were determined to be eligible and enrolled (Figure 1).

In hindsight, it became clear that each patient satisfied the criteria for the standard AS classification [2,17]. The average age of the patient was 31.2 years (SD = ±9, range = 19-56) years, and the disease had been present for 99.5 months (SD = ±67.8, range = 13-312) months. Human leukocyte antigen (HLA) B27 was found in 86% of patients. According to medical records, all patients had taken multiple courses of more than two NSAIDs, often under supervision, in the recent past. Table 1 shows the use and consumption of NSAIDs.

**Table 1:** Efficacy variables (mean ± SD) at baseline and predetermined study time points in a study of biosimilar adalimumab (administered for the initial 12 weeks) in symptomatic ankylosing spondylitis (n = 50 patients) (95% confidence interval shown in parenthesis). Note: see text for methods. All numerical values rounded to one decimal place *: Significant difference from baseline at p <0.05 analysis of variance (ANOVA), intention-to-treat analysis VAS: Visual Analog Scale; PATG: patient global assessment; HAQ: Health Assessment Questionnaire (Indian version); ESR: erythrocyte sedimentation rate (Westergren, mm fall end first hour); CRP: C-reactive protein (mg/L)

Variable/Time points	Baseline	Week 4*	Week 8*	Week 12*	Week 24	Week 36	Week 48*
Pain VAS	5.9 ± 2.0 (5.3, 6.5)	3.7 ± 1.7 (3.3, 4.2)	3.2 ± 1.9 (2.6, 3.7)	2.6 ± 2.1 (2, 3.2)	3 ± 2.4 (2.3, 3.7)	3.6 ± 2.4 (2.9, 4.2)	3.92.2 (3.3, 4.6)
PATG	6.2 ± 1.6 (5.7, 6.6)	4 ± 1.5 (3.6, 4.5)	3.3 ± 1.6 (2.9, 3.8)	2.9 ± 1.9 (2.3, 3.4)	32 (2.6, 3.5)	3.5 ± 2.1 (2.8, 3.9)	3.72.3 (3.1, 4.4)
HAQ	5.3 ± 3.7 (4.2, 6.3)	3.1 ± 2.8 (2.3, 3.9)	2.5 ± 2.4 (1.8, 3.1)	2.1 ± 2.8 (1.4, 3)	3 (2.2, 3.9)	3.5 ± 3.2 (2.6, 4.4)	3.4.4 (2.5, 4.4)
ESR	88 ± 30 (80, 97)	29.1 ± 20 (23.4, 34.8)	31.4 ± 20 (23.4, 34.8)	33.929.1 (25.6, 42.1)	54 ± 33.2 (43.4, 62.3)	69.837.2 (59.3, 80.5)	68.5 ± 33.2 (59, 78)
CRP	63.8 ± 55.2 (48.1, 79.5)	9.6 ± 16.8 (4.8, 14.4)	13.2 ± 29.6 (4.8, 21.6)	10.9 ± 21.4 (4.8, 17.0)	25.0 ± 39.4 (13.8, 36.2)	35.0 ± 44.8 (22.3, 47.8)	35.6 ± 38.0 (24.8, 46.4)

A total of 28 patients provided a history of supervised long-term (at least six months) DMARD (sulfasalazine and/or methotrexate) use. Pain VAS and other individual variables revealed that patients had moderately severe symptomatic active disease (Table 2).

**Table 2:** Proportion percentage (95% confidence intervals) of patients showing ASAS 20 (primary efficacy response), ASAS 40, ASAS 5/6, ASAS partial remission, and mean of ASDAS (ESR), BASDAI, and BASFI indices at predetermined study time points in a study of biosimilar adalimumab (administered during the initial 12 weeks of study) in ankylosing spondylitis (n = 50 patients): a single-arm prospective observational study (95% confidence interval, intention-to-treat analysis). ASAS: Assessment Spondyloarthritis International Society; ASAS 20: least 20% improvement in three of the four domains - patient global assessment, overall pain, function (BASFI), and inflammation (mean of BASDAI questions of morning stiffness), and no worsening; ASAS 40: least 40% improvement in the ASAS 20 domains and no worsening; ASAS 5/6: 20% improvement in ASAS 20 domains, lateral spine flexion, C-reactive protein assay; ASAS partial remission: all four ASAS 20 domains score less than 2 on numeric scaling rate (NSR); ASDAS: a weighted index of disease activity - total back pain, patient global disease activity, peripheral painful/swollen joint, morning stiffness and erythrocyte sedimentation rate; BASDAI: Bath Disease Activity Index; BASFI: Bath Functional Index-10 items

Study visits (weeks)	ASAS 20	ASAS 40	ASAS 5/6	ASAS 20 partial remission	ASDAS	BASDAI	BASFI
0 Baseline	-	-	-	-	4.6 (4.4, 4.9)	5.3 (4.7, 5.8)	4.7 (4, 5.3)
4	56 (42.3, 68.8)	28 (17.5, 41.7)	38 (25.9, 51.8)	4 (1.1, 13.5)	2.6 (2.4, 2.9)	3 (2.5, 3.5)	3.1 (2.5, 3.8)
8	74 (60.4, 84.1)	58 (44.2, 70.6)	64 (50.1, 75.9)	16 (8.3, 28.5)	2.4 (2.1, 2.7)	2.3 (1.9, 2.7)	2.4 (1.8, 3)
12	82 (69.2, 90.2)	70 (56.2, 80.9)	68 (54.2, 79.2)	34 (22.4, 47.8)	2.4 (2.1, 2.6)	1.8 (1.3, 2.2)	2 (1.4, 2.6)
24	68 (54.2, 79.2)	42 (29.4, 55.8)	54 (40.4, 67)	30 (19.1, 43.8)	2.9 (2.6, 3.2)	2.4 (2, 2.6)	2.6 (2, 3.3)
36	54 (40.4, 67)	34 (22.4, 47.8)	40 (27.6, 53.8)	22 (12.8, 35.2)	3.1 (2.7, 3.4)	2.6 (2.1, 3.1)	2.7 (2, 3.4)
48	5 2(38.5, 65.2)	28 (17.5, 41.7)	58 (44.2, 70.6)	22 (12.8, 35.2)	3.5 (3.2, 3.8)	2.7 (2.1, 3.8)	3 (2.2, 3.7)

According to the BASDAI questionnaire, the baseline mean spine pain was 6.8 ± 2.1 (SD) (range = 0-10 cm). ASDAS classified 42 (84%) patients as having a very high disease activity [14]. Eleven patients withdrew, with the majority withdrawing due to a poor therapeutic response. There were no AE-related withdrawals. After 30 weeks of observation, one patient experienced hip pain that became intolerable and ultimately required arthroplasty. The quantitative use of NSAID (etoricoxib) is shown in Table 3.

**Table 3:** NSAID (etoricoxib or etoricoxib equivalent) consumption during the trial study of biosimilar adalimumab (Bs-ADA) in the study of patients (n = 50) with symptomatic moderately severe chronic ankylosing spondylitis: a single-arm prospective observational study. Note: Bs-ADA was injected using standard regimen for 12 weeks beginning at baseline NSAID: non-steroidal anti-inflammatory drug; mean daily dose pertains to etoricoxib (Nucoxia™/Ezycoxib™ available as 60 mg and 90 mg tablet, India pharmacopeia); over 90% of patients consumed etoricoxib in the study; dose of any other NSAIDs converted empirically to etoricoxib equivalent; *: minus prefix indicates period prior to baseline; 48+; study completion and a 12-week follow up; **: either od or bid, 60-120 mg etoricoxib; ***: 60 mg etoricoxib od on as-needed basis; ^: advised need basis but did not require any NSAID use. See text for details, especially for etoricoxib equivalent.

Weeks, 0 Baseline/Variables	0 to 4*	0 to 2	2 to 4	4 to 6	6 to 8	8 to 12	12 to 24	24 to 36	36 to 48	48+
Number of patients	50	50	50	49	49	49	49	47	44	39
Mean daily dose mg (SD)	92.4 (27.62)	104.7 (25.3)	108.7 (31)	77.3 (46.7)	35.1 (42.4)	19.6 (35.7)	17.2 (32,2)	22.2 (38.9)	26.6 (42.8)	50.5 (50.1)
**Continuous use	50	50	45	37	17	9	4	6	9	17
***As-needed basis	0	0	5	7	13	15	14	15	19	11
^Nil use	0	0	0	5	19	25	31	26	16	11

Other NSAIDs were taken by nine patients (three patients took indomethacin, three patients took naproxen, two patients took diclofenac sodium, and one patient took etodolac; their doses were converted to etoricoxib equivalents). After two weeks, the mean pain VAS was 4.2 ± 2 (SD). However, after four weeks of study, the use of NSAIDs was significantly reduced (three Bs-ADA injections). After eight weeks, fewer than 15% of patients required NSAIDs on a daily basis. At week 12, four of the nine patients who took NSAIDs on a daily basis showed an ASAS 20 index response.

**Figure 2:**
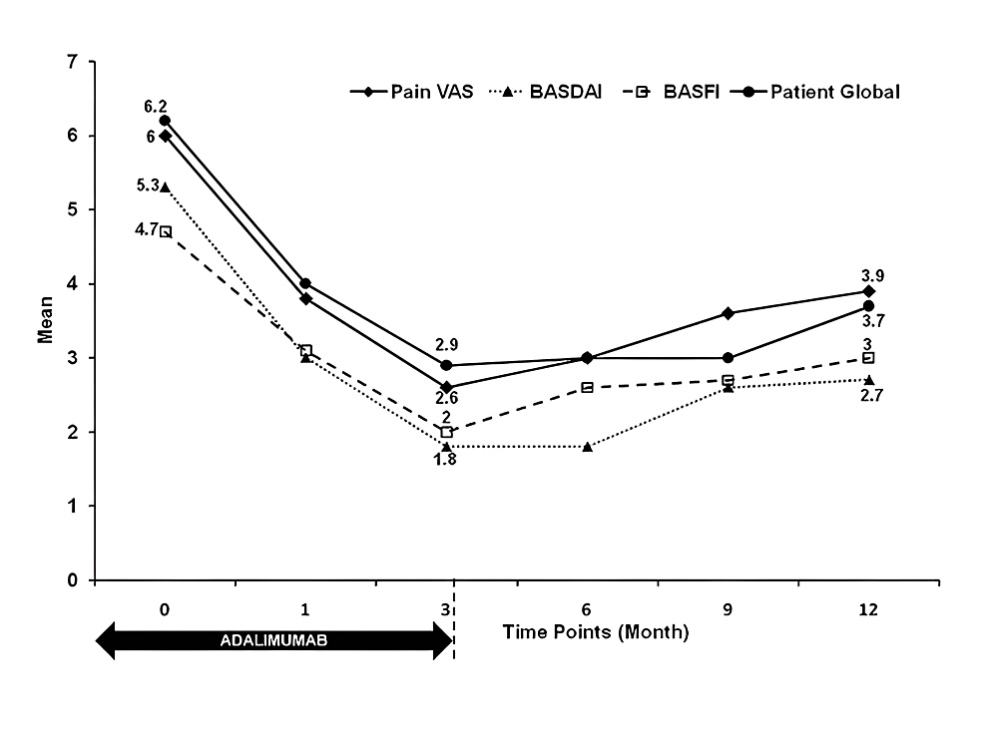
Over time selected efficacy measures (mean): an open-label study of biosimilar adalimumab in ankylosing spondylitis (n = 50 patients). VAS: Visual Analog Scale; BASDAI: Bath Ankylosing Spondylitis Disease Activity Index; BASFI: Bath Ankylosing Spondylitis Functional Index

Table 2 shows that the ASAS 20 improved in 41 (82%) patients (95% CI = 69.2%, 90%) and the ASAS 40 improved in 35 (70%) patients (95% CI = 56.2%, 80.9%). At all primary efficacy time points, more than half of the patients achieved an ASAS 20 response. Proof of concept was provided by 21 patients (42%) who showed a continued improvement on the ASAS 20 index at the week 12, 24, and 36 time points (see statistical design and data analysis). Patients improved significantly with early response, which persisted, albeit with some decline, until the study’s completion at 48 weeks (one year) (Table 2, Figure 2), with week 12 showing the greatest improvement.

According to ASDAS, 28 (56%) patients were classified as having inactive/low disease activity at week 12, 18 (36%) patients at week 24, 12 (24%) patients at week 36, and 10 (20%) patients at week 48 endpoint; correspondingly, a major improvement was observed in 64%, 50%, 36%, and 28% of patients, respectively [14]. In the cohort within the first 4-12 weeks of study, ASAS 20 attained a high cumulative proportion (~80%), as shown in Table 4.

**Table 4:** Survival analysis: displays the cumulative proportion of people who survive a specific event (ASAS Index) at the end of a predetermined study visit. Interval (weeks), standard error (in parenthesis), and 95% confidence interval in a one-year observational study on short-term (12 weeks) use of biosimilar adalimumab in 50 patients with active chronic ankylosing spondylitis. Note: The selected ASAS index qualified as an event for the survival analysis if it persisted for at least eight weeks after the first occurrence at the predetermined study time points ASAS: Assessment Spondyloarthritis International Society; ASAS 20: least 20% improvement in selected domains (patient global assessment, overall pain, function, and inflammation); ASAS 40: least 40% improvement in the ASAS 20 domains and no worsening; ASAS 5/6: 20% improvement in ASAS 20 domains, lateral spine flexion, C-reactive protein assay; ASAS partial remission: all the four ASAS 20 domains score less than 2 on numeric scaling rate (NSR)

Predetermined study visit time points (weeks)	ASAS 20		ASAS 40		ASAS 5/6		ASAS partial remission	
	Mean (SD)	Range	Mean (SD)	Range	Mean (SD)	Range	Mean (SD)	Range
4	0.50 (0.07)	0.36, 0.63	0.78 (0.06)	0.66, 0.89	0.72 (0.06)	0.59, 0.84	0.98 (0.02)	0.94, 1.01
8	0.28 (0.06)	0.15, 0.4	0.6 (0.07)	0.46, 0.73	0.54 (0.07)	0.4, 0.67	0.90 (0.04)	0.81, 0.98
12	0.20 (0.06)	0.15, 0.31	0.54 (0.07)	0.4, 0.67	0.44 (0.07)	0.3, 0.57	0.74 (0.06)	0.61, 0.86
24	0.18 (0.05)	0.15, 0.28	0.52 (0.07)	0.38, 0.65	0.44 (0.07)	0.3, 0.57	0.72 (0.06)	0.59, 0.84
36	0.16 (0.05)	0.15, 0.26	0.48 (0.07)	0.34, 0.61	0.42 (0.07)	0.28, 0.55	0.68 (0.07)	0.55, 0.8
48	0.16 (0.05)	0.15, 0.26	0.48 (0.07)	0.34, 0.61	0.42 (0.07)	0.28, 0.55	0.68 (0.07)	0.55, 0.8

Both the ASAS 40 and the ASAS 5/6 showed a pattern (time to event) that was very similar; however, the cumulative proportion was significantly less (less than 50%), and the curve remained nearly flat (with minimal increment) after that point. The time to event for the optimal (~30%) cumulative proportion for an ASAS partial remission event was later at 12-24 week intervals and remained nearly flat thereafter (week 36: 95% CI = 20%, 45%). This was found to be the case when the patients were observed at intervals ranging from 12 to 24 weeks. Seven patients examined at week 12 did not achieve an ASAS 20 response. Even though each of the non-responders was given an additional two injections of Bs-ADA (see methods), not one of them achieved an ASAS 20 response after that point.

**Figure 3:**
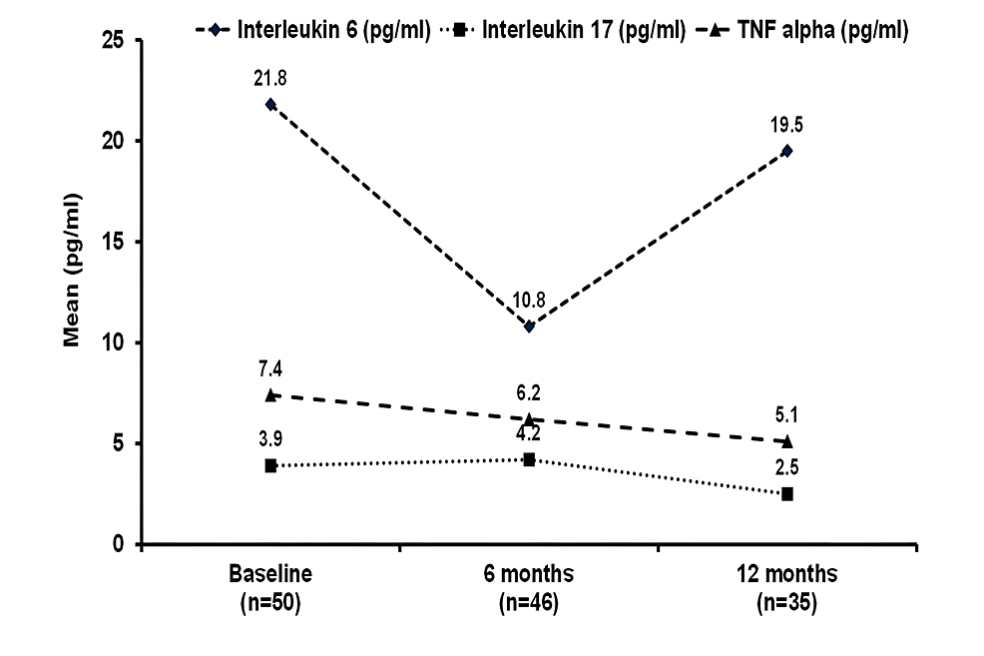
Selected cytokine assay (mean) over time: a biosimilar adalimumab open-label study in ankylosing spondylitis (n = 50 patients). TNF: tumor necrosis factor

There were no serious adverse events reported by any of the patients. There were no fatalities. No patient developed symptoms of TB. Ten patients experienced mild AEs, which were resolved with standard care; however, none of the patients had a local site reaction (three patients with nasopharyngitis, three patients with dermatophytosis, two patients with non-specific body aches and pains, two patients with mild uveitis, one patient with nasal polyp bleed, one patient with itchy eczema over ankle region). Figure 3 shows a reduction in pro-inflammatory cytokines assay (IL-6, TNF, and IL-17) at weeks 24 and 48 compared to baseline, which was significant for IL-6 assay at week 24 (p < 0.05).

## Discussion

After receiving treatment with biosimilar adalimumab over 10 weeks, patients with active chronic symptomatic AS showed an early onset of significant improvement in this prospective study that lasted a year (six injections). Before enrolment, patients had a poor response to NSAIDs, and their consumption was significantly reduced but continued for a long time (Table 1). After stopping adalimumab, several variables and indices continued to show significant and substantial improvement (Table 2). At week 12, 82% of patients displayed an improvement in the ASAS 20 index (primary efficacy), and more than 50% of patients continued to respond for at least 24 weeks. Eleven patients discontinued treatment due to insufficient improvement. Every AE was mild. The study was conducted in an actual community practice setting.

It is challenging to determine how much improvement a patient with chronic AS wants. This study used a number of standard index responses (ASAS, BASDAI, BASFI, and ASDAS) to demonstrate a multifaceted improvement [2]. According to the survival curves, these indices also showed persistence of significant improvement over the course of the study. However, with ASDAS, the improvement was noticeably less. In our experience, patients frequently find it difficult to quantify morning stiffness, and we think that some study participants were unable to donate blood in a fasting state due to challenging personal logistics.

The ASAS partial remission in this study was oddly 34% at week 12, and it largely persisted after stopping adalimumab (Table 2). In chronic AS, remission is hard to define [18]. According to reports, up to one-third of patients receiving NSAIDs and up to two-thirds receiving anti-TNF agents are reported to experience an ASAS partial remission, with patients experiencing a higher response in cases of more recent disease [18]. In anti-TNF studies, partial remission (ASAS) was reported in 1.03-5.6% of patients on placebo, indicating the rarity of spontaneous remission in AS [18].

Anti-TNF drugs should be used in AS long-term, according to current recommendations. For example, after taking infliximab regularly for three years, several patients experienced relapses within six months of stopping it [19]. However, after receiving six injections of Bs-ADA in this study, a number of patients continued to experience symptomatic improvement (Table 2 and Figure 2) with good function (low BASFI) for at least 24 weeks. In some patients who are struggling with AS, short-term therapy (anti-TNF) might be administered cyclically (10 weeks of therapy every 24 weeks) with good efficacy and safety. However, appropriate long-term clinical studies must be conducted to validate this.

Other drawbacks and advantages existed as well. The study had an uncontrolled design, a single center, and a small sample size. The study’s fundamental idea was motivated by a therapeutic need and clinical observations [5]. While patients were managed in a community setting and demonstrated good compliance, the protocol was pragmatic in nature, as evidenced by the fact that six of the 11 withdrawals occurred after the study’s initial 36 weeks. The experience had an impact on a number of the current selection criteria and methods, which were modified from regulatory drug trial studies and standard recommendations (ASAS) [3,20]. The overall result must have been impacted, particularly in the beginning, by the placebo effect brought on by the patients’ expectations of Bs-ADA. The benefit of long-term anti-TNF treatment on AS bony lesions appears to be modest, and the current study did not assess anti-adenosine deaminase (ADA) antibodies or radiological (structural) changes [21]. This study complied with the standards for rheumatology’s longitudinal observational drug studies recommended by the European Union League Against Rheumatism (EULAR) [22].

Standard treatment for AS involves the continued use of NSAIDs, and a positive symptomatic response is typically anticipated within two weeks [3]. A one-year randomized controlled trial of a daily fixed dose of etoricoxib demonstrated excellent efficacy and safety in patients with AS, despite the fact that the disease duration was not specified. The 90 mg and 120 mg groups had nearly identical results [23]. Before participation, 77.4% of patients experienced a significant flare-up following the washout phase. The pain reduction was significant after two weeks and lasted until the study was completed (one year). The ASAS 20 index improvement at week six was 64.8%, and the ASAS partial remission was around 15%. In another randomized controlled trial that lasted for 24 weeks, the combination of infliximab and naproxen proved to be significantly more effective than naproxen on its own. Patients in both groups experienced significant early pain relief and a sustained decrease in BASDAI and CRP. The duration of the disease was less than three years [24].

Patients in this cohort had active chronic AS and did not respond well to NSAIDs. In our clinic, the preferred NSAID for AS treatment is etoricoxib. Despite previous regular daily use, the pain at the start (week 0) was moderately severe (Table 2). Patients, however, continued to take etoricoxib at the therapeutic dose in addition to Bs-ADA. The reduction in pain after four weeks (-2.2, 95% CI = -2.77, -1.65) was substantial and significant (Table 2), and there was a significant and persistent reduction in the need for NSAIDs (Table 1). After week 10, patients were no longer taking Bs-ADA. During the first 12 weeks, fewer than 15 patients admitted to using oral paracetamol and tramadol on an as-needed basis (data not shown). The severity of active painful disease was comparable between this study and the two NSAID drug trials that were described earlier, despite the significant differences in selection criteria and study design.

The current research did not include a washout period at any point. In this investigation, NSAIDs (etoricoxib) provided less pain relief. Despite being reasonably sure of the data on NSAID use shown in Table 1, it cannot be vouched. It highly depended on patient compliance and recall, which are notoriously difficult to evaluate. Data on NSAID use daily and absent use are likely more reliable (Table 1). There is a possibility that etoricoxib is not the most effective NSAID for every individual. We frequently encounter patients (and members of the community) who express intense fear of taking NSAIDs and analgesics due to the potential for life-threatening side effects, and our expert counseling frequently fails. Nonetheless, a large number of deserving AS patients cannot afford biologic agents, despite their desire to use them. The findings of this study should also be weighed against this community perspective as well.

In a randomized active-controlled drug trial study, the ASAS 20 index response at week 24 was 75% in the group that received the biosimilar version of adalimumab (IBI1303) and 72% in the group that received the original version of adalimumab (Humira™) [25]. In this uncontrolled study, the ASAS 20 index response at week 24 (intention to treat) was 68%; however, patients in this study did not receive Bs-ADA for more than 10 weeks.

After 24 weeks of use, a recent study that was conducted in an AS registry in India found that Bs-ADA (Exemptia™) significantly improved both the pain VAS and the BASDAI. However, only one-third of the patients had sufficient data for the analysis to be performed [26]. TB was not reported by any of the patients in the Indian registry. In some Indian studies, 124 patients with ulcerative colitis and Crohn’s disease received prolonged treatment with Bs-ADA (Exemptia™), and nine developed TB [27]. Six patients were excluded from this study after testing positive for latent TB during the screening process. The aggressive use of TB screening appears to have reduced the incidence rate of TB from 1.5 to 0.2 per 100 patient-years in adalimumab drug trials for the treatment of rheumatic disorders [28].

The assay for serum TNF showed a progressive, albeit modest, reduction in levels between weeks 24 and 48; similarly, the assay for IL-17 showed a modest rise and then fall in levels. All of these results were compared to the baseline reading (Figure 2). On the other hand, IL-6 showed a significant decrease at week 24 (which is consistent with the CRP assay in Table 2). The assay was much higher at week 48, likely reflecting a rise in CRP and a noticeable decline in clinical improvement. Serum pro-inflammatory cytokine assays have been reported in spondyloarthritis patients treated with anti-TNF agents, but this is not standard care [29].

Immense clinical improvement was seen during the management of AS (CRD) patients while using anti-TNF agents for a short period of time (12-16 weeks) with similar clinical improvements without encountering a single case of TB. Patients in desperate need of anti-TNF agents but with a positive test for latent TB were invariably given a six-month course of INH prophylaxis.

This article was previously posted to the medRxiv preprint server on November 15, 2021 [30].

## Conclusions

The study found that a 10-week standard regimen (six injections) of Bs-ADA (Exemptia™) produced remarkable clinical improvement in patients with symptomatic active chronic AS who did not respond to NSAIDs. After discontinuing the Bs-ADA, several patients’ clinical responses lasted at least 24 weeks. It was found that the treatment was well tolerated overall and was risk-free. Short-term use of Bs-ADA is viable for treating difficult AS in resource-constrained settings like ours. However, more research is needed before it can be accepted as a standard treatment modality for AS.
